# Genetic Map Construction and Quantitative Trait Locus (QTL) Detection of Growth-Related Traits in *Litopenaeus vannamei* for Selective Breeding Applications

**DOI:** 10.1371/journal.pone.0075206

**Published:** 2013-09-25

**Authors:** Farafidy Andriantahina, Xiaolin Liu, Hao Huang

**Affiliations:** 1 College of Animal Science and Technology, Northwest A&F University, Shaanxi Key Laboratory of Molecular Biology for Agriculture, Yangling, Shaanxi, People’s Republic of China; 2 Hainan Guangtai Ocean Breeding Company Limited, Haikou, People’s Republic of China; Auburn University, United States of America

## Abstract

Growth is a priority trait from the point of view of genetic improvement. Molecular markers linked to quantitative trait loci (QTL) have been regarded as useful for marker-assisted selection (MAS) in complex traits as growth. Using an intermediate F_2_ cross of slow and fast growth parents, a genetic linkage map of Pacific whiteleg shrimp, *Litopenaeusvannamei* , based on amplified fragment length polymorphisms (AFLP) and simple sequence repeats (SSR) markers was constructed. Meanwhile, QTL analysis was performed for growth-related traits. The linkage map consisted of 451 marker loci (429 AFLPs and 22 SSRs) which formed 49 linkage groups with an average marker space of 7.6 cM; they spanned a total length of 3627.6 cM, covering 79.50% of estimated genome size. 14 QTLs were identified for growth-related traits, including three QTLs for body weight (BW), total length (TL) and partial carapace length (PCL), two QTLs for body length (BL), one QTL for first abdominal segment depth (FASD), third abdominal segment depth (TASD) and first abdominal segment width (FASW), which explained 2.62 to 61.42% of phenotypic variation. Moreover, comparison of linkage maps between *L* *. vannamei* and *Penaeus* *japonicus* was applied, providing a new insight into the genetic base of QTL affecting the growth-related traits. The new results will be useful for conducting MAS breeding schemes in *L* *. vannamei* *.*

## Introduction

Increased market demands for penaeid aquaculture production has determined the progress of genetic improvement approaches focused on both higher productivity and reduction of rearing costs [1]. A major challenge in current biology is to understand the genetic basis of variation for quantitative traits [2]. Quantitative traits are determined by the combined forces of genetic and environmental factors and also the interaction between them [3]. Understanding the relationship between variations in DNA sequences and variations in phenotypes for these quantitative traits will yield insights that are important for enhancing the speed of selective breeding schemes in agriculturally important plants and animals and for predicting adaptive evolution [2].

*Litopenaeusvannamei* (Pacific whiteleg shrimp) is one of the most important shrimp species farmed worldwide with important values in aquaculture [4]. *L* *. vannamei* is also one of the shrimp species with the most extensive selective breeding practices in the world. However, its quantitative trait loci (QTL) associated to growth-related traits has been rarely identified. Growth-related traits (for instance, body weight and body sizes), which are economically important complex traits, have been studied in the genetic improvement researches [5,6]. Understanding the genetic basis of these complex traits is important to better conduct the genetic improvement schemes. The most direct technique to identify these loci is by fine-mapping QTL and identifying positional candidate genes, and to conduct maker-assisted selection (MAS) or gene-assisted selection (GAS). To date, genetic mapping of penaeid species has been slow and concentrated mostly on a few important cultured species such as *Penaeus* *monodon* [7], *P* *. japonicus* [8], *L* *. vannamei* [9], and *P* *. chinensis* [10] using primarily dominant amplified fragment length polymorphism (AFLP) markers and a few codominant simple sequence repeats (SSR) or microsatellite markers. However, no precise QTL associated to growth-related traits have been identified based on *L* *. vannamei* linkage maps. Conducting genetic map construction and QTL analysis are very useful in improving the speed of genetic improvement and employing MAS in *L* *. vannamei* .

AFLP markers were reported to show a greater level of polymorphism and informativeness than any other markers [11], and have been widely used in linkage mapping of several shrimp species [7–10]. SSR markers have been increasingly used in the construction of linkage maps in the past decade for aquatic species [10,12–14]. In this research, we constructed a genetic linkage map of *L* *. vannamei* with AFLP and SSR markers using an intermediate F_2_ cross of slow and fast growth parents and identified 14 QTLs associated with growth-related traits in *L* *. vannamei* . With the object of gaining new understanding of genes related to growth traits in a commercial penaeid species, the specific objectives of this present research were to determine the loci that are responsible for variation in growth-related quantitative traits and to provide insight into the genetic architecture of the traits that are interesting for breeding applications in *L* *. vannamei* *.*

## Materials and Methods

### Animal materials

The parent stocks of *L* *. vannamei* used in this research were obtained from a commercial pond of Hainan Guangtai Ocean Breeding Company Limited (Wenchang Breeding Base), China. Following spawning and larval culture, approximately 20,000 progeny were stocked into a 666.6 m^2^ (33.33 m × 20 m × 1.5 m) pond at an initial density of 30 per m^2^ for commercial growout. Animals were harvested after about 6 months of pond-growth. At the harvest, broodstock were selected to establish the laboratory-cultured lines for our research. During harvest, a sample of shrimp was measured for determining the size distribution of pond stocks and weight threshold for selection. From these shrimp, five males and 25 females were chosen as founder parents of slow-growth (SG) line; the same number of shrimp was used to initiate fast-growth (FG) line A further 20 FG and SG males and 20 FG and SG females were also sampled to make crosses between FG and SG parents. After selection, the shrimp were transferred to an experimental laboratory. They were measured for total length (TL), body length (BL), first abdominal segment depth (FASD), third abdominal segment depth (TASD), first abdominal segment width (FASW) and partial carapace length (PCL), using a digital camera and Photoshop software [5]. Shrimp body weights (BW) were determined using an electronic balance. An intermediate F_2_ full-sib population of 43 shrimp, produced by reciprocally mating 4 FG and SG grandparents randomly selected from the above populations and one F_1_ FS maternal and one F_1_ SF paternal parent, were used for map construction. Seven growth-related phenotype traits that were measured according to Andriantahina et al. [5] including BW, TL, BL, FASD, TASD, FASW and PCL, were measured for QTLs identification.

### AFLP analysis

DNA was isolated from the abdominal muscles of *L* *. vannamei* using a standard phenol–chloroform method [15]. AFLP analysis was performed according to the protocols described by Vos et al. [16] with small modification. Digestion–ligation reaction mixture contained 3 µl genomic DNA (about 200 ng), 1.1 µl 10 x T4 DNA ligase buffer with ATP, 1.1 µl 0.5 M NaCl, 0.55 µl of 1 mg/ml bovine serum albumin (BSA), 1 U *Mse*I, 5 U *EcoR*I, 1.0 U T4 DNA ligase, 50 pmol *Mse*I adaptor, 5 pmol *EcoR*I adaptor and water to bring the final volume to 11 µl. The reaction mixture was incubated at 37 °C for 2 h and then diluted with 150 µl TE_0.1_ (20 mM Tris–HCl, 0.1 mM EDTA, pH 8.0). Preselective amplification was carried out using primers complementary to adaptor sequence without any additional nucleotides at 3’ end. Selective primers consisted of preselective primers with three selective nucleotides added to 3’ end. *Eco*RI-selective primers were fluorescently 5’ labeled with FAM. PCR reactions were performed in a PTC-100 or PTC-200 thermal cycler (MJ Research). PCR products labeled with FAM dye were analyzed with an ABI Prism 310 sequencer.

AFLP markers were named according to the primer pairs used to generate them and also their size. *Eco*RI- and *Mse*I-selective primers were respectively coded by letters and numbers followed by a letter f (fragment) and digits representing the size in base pairs [17]. For instance, D8f100 refers to 100-bp fragment generated by *Eco*RI primer D (ACG) and *Mse*I primer 8 (CAT).

### Microsatellite genotyping

Microsatellites were scored using 0.2 µM dye-labeled (5-FAM) forward primers and 0.5 µM reverse primers. PCR reactions were conducted as follows: initial denaturation for 5 min at 94 °C, followed by 35 cycles for 30 s at 94 °C, 30 s at optimal annealing temperature, and 30 s at 72 °C, last followed by 10 min at 72 °C. PCR products labeled with FAM dye were also analyzed with an ABI Prism 310 sequencer.

We tested 147 microsatellite loci recently described by Meehan et al. [18] and Ball et al. [19] for this research work, of which 25 SSR markers were informative. Because most of the original name is too long to be permitted when using mapping software MapMaker/Exp v. 3.0, SSR markers were renamed according to information of the species, trial order in our analysis, and the size of the microsatellite bands. The first letter of marker indicates the species (e.g., v for *L* *. vannamei* ), followed by digits representing analyzing order in our work and the size of microsatellite band similar with AFLP markers. For example, SSR marker v1f148 indicates that SSR marker of *L* *. vannamei* was analyzed first in our trials with a size of 148 bp.

### Linkage analysis and genome coverage

Genotype data was recorded in a TXT file and imported to Mapmanager QTXb20 software [20]. A Chi-squared test was used to assess Mendelian segregation distortion of all polymorphic loci data in F_2_ population before linkage analysis. The data set was designated as “intercross” and linkage groups were assigned with *P*-value of 1.0E-4 by using the “Make linkage groups” command and then linkage groups were accordingly adjusted by the “Distribute” and “Ripple” command. The consensus linkage map from AFLP and SSR markers was graphically represented using MAPCHART2.1 program [21] based on calculated map distances between markers.

Two approaches were used to assess genome length according to Liu et al. [10]. Under G_e1_ the genome length was assessed by adding 2 s (where s is average space of linkage map) to the length of each group, which accounts for chromosome ends. Under G_e_, the genome length was estimated by multiplying the length of each linkage group (LG) by (m+1) / (m−1), where m is the number of markers in each LG. The estimated map length is the sum of revised lengths of all LGs [22]. The average of these two estimates was used as estimated genome length (G_e_). The observed map length was calculated as total length of map (G_of_) excluding triplets and doublets (LGs with three and two markers, respectively) or as total length of map including the triplets and doublets (G_oa_). The observed genome coverage, C_of_ and C_oa_, was calculated as G_of_/G_e_ and G_oa_/G_e_ respectively [23].

### QTL analysis

First, the normality of growth-related quantitative data was estimated using a one-sample Kolmogorov-Smirnov test implemented in SPSS 17.0 package, no deviations from a standard normal distribution were found for the studied traits. QTL analysis was performed by means of composite interval mapping (CIM) [24] implemented in Windows QTL Cartographer 2.5 software [13] using 1000 permutations with significance as 0.05. A minimum limit of detection (LOD) threshold of 3.0 was used for considering a significant QTL, and the percentage of phenotypic variance explained by each QTL was assessed using Windows QTL Cartographer 2.5.

### Comparative mapping

Comparative genomics can provide valuable information about the architecture and functional organization of a species’ genome [25]. A total of 25 AFLP loci placed on the linkage map were used to perform comparative mapping analysis with *P* *. japonicus* , as the flanking regions of AFLP markers have the ability to identify chromosomal regions that are homologous across species [11,26]. The total genome of *P* *. japonicus* has been sequenced and is closely related to shrimp on phylogenetics. The flanking sequences of 25 *L* *. vannamei* AFLP loci were used as queries to blast against the *P* *. japonicus* whole genome data through National Center of Biotechnology Information (NCBI) (http://www.ncbi.nlm.nih.gov/genome/).

## Results

### Polymorphism and segregation of molecular markers

A total of 100 AFLP primer combinations in F_2_ population produced approximately 3158 AFLP bands. On average, each primer combination produced 40 to 80 bands with a size between 10 and 1200 bp. Among the 3158 bands obtained, 600 were polymorphic accounting for 19% of the total (Table 1). An average of eight polymorphic markers was detected per primer. Variation was evident in the number of polymorphic fragments revealed by different primer combinations. The level of polymorphism produced by different primer combinations varied considerably from 2% to 20%. Of the SSR markers genotyped, 25 markers were informative in mapping population of *L* *. vannamei* and available for map construction (Table 2).

**Table 1:** Numbers of polymorphic markers generated by 100 *Mse*I and *Eco*RI AFLP primer combinations.

***Mse*I**	***Eco*RI**															
	**AAG(A**)	**ACA(B**)	**ACA(C**)	**ACG(D**)	**AGC(E**)	**AGG(F**)	**CCG(G**)	**CCT(H**)	**CTC(I**)	**TAC(J**)	**ACG(K**)	**ATA(L**)	**AAA(M**)	**ACC(N**)	**AGG(O**)	**Total**
ACA:1			4		1	4		1	4	2	1	3	3	1	3	27
ACC:2		2			3	3				3	2	1	3	2	4	23
ACT:3		3	4	1	3	1			3		1	2	2	1		21
ATC:4	5		4		1	1			3			2	2	1	3	22
CAA:5	6		3	2	3	4	3		2	4		1	3	1	2	34
CAC:6	5				4	1	2			3		2	1	1	2	21
CAG:7	6	5	3	2	3	3	2	4	2		1	2	3	1		37
CAT:8	6	3	4	4	3	3	3	1	3	3	2	1		1	2	39
CCA:9	6	1	3	3	1	4	1	3	4	1	3	2	2	1		35
CCG:10	3	4	4	3	4	1	3	1	2		2	1	3	1	3	35
CCT:11	3	1	3	1	1	1	1	2	3		1	1	1	1		20
CGA:12		1	3	1			1	2	3		3	2	3	1	3	23
CGC:13		3	1	3		2	1	1	3		1	1		1	1	18
CGG:14		1		2		4	1	1			1	2		1	2	15
CGT:15		3		1	4	4	1	4			1	2	2	1	1	24
CTA:16	1	1	2	3	5		2	1	3	3	1	2	2	1	1	28
CTC:17	2	1	4	4	4	5	3	3	3	3	1	1	2	1		37
CTG:18		1	2	4			2	4	3		1	2	3	1	3	26
CTT:19	4		2	1		4	3	5	2	5	1	3	2	1		33
GAA:20				1				1		4	1	1	2	1	3	14
GAC:21	1							1			1	2	3	1		9
GCC:22	1		1					1	2	3	1	2		1	1	13
GTA:23			2						3	3	2	1		1	1	13
TAA:24											1		2	1	2	6
TAC:25	2										2				2	6
TAG:26	5									2	1					8
TCA:27	3									4	1					8
TGA:28	2										1		2			5
Total	61	30	49	36	40	45	29	36	48	43	34	39	46	25	39	600

**Table 2:** Informative microsatellite markers in *L* *. vannamei* ^**a**^.

**Renamed Markers**	**Corresponding markers in original conference**	**Primer sequences (5’–3’**)	**T_a_ (^0^C**)	**GenBank number**
v2	Lv8.220	CGAGTGGCAGCGAGTCCT	57	AF360072
v6	Lv6.23	TATTCCCACGCTCTTGTC	60	AF360031
v9	Lv9.43	CCTTGACACGGCATTGATTGG	60	AF360109
v26	Lv5.45	TACGTTGTGCAAACGCCAAGC	54	AF360025
v29	Lv7.56	TGC TGAAGCTACACTACCTTCG	62	AF360055
v43	Lv8.256	CACATGCCTTTGTGTGAAAACG	55	AF360076
v90	Lv9.103	ACACTCACTTATGTCACACTGC	55	AF360090
v92	Lv10.33	TACACACCAACACTCAATCTCC	55	AF359992
v98	Lv10.312	TGTTAGGAATGCTTATGA	59	AF359989
V110	Pse036	AGCCAATTAGACAGGTAG	55	AF047361
v113	Lv5.35	GATAGAGAGGTCAACAAACG	52	AF360023
v114	Lv6.63	CCTTCGTCGTCCTCTTACTA	55	AF360040
v117	Lv7.97	TGGAAGAAACTAAGAGAGCA	59	AF360057
v119	Lv8.9	CTTTATCTACACACAAACCGC	55	AF360089
v120	Lv8.176	TGATACCAAAGGCTGTAGAGG	50	AF360063
v122	Lv9.28	TCTGGAAAGAAATGAAAGT	59	AF360108
v123	Lv9.60	AATACAACAATCCTTTAGTC	50	AF360111
v126	Lv9.145	TTCTTCGCCAGGAAACAG	55	AF360098
v129	Lv9.174	CGCGGTCACACAAGCATA	59	AF360104
v130	Lv9.178	GAGAAATTCGATTATCAG	55	AF360105
v133	Lv10.27	CGCTAAGTTATGCTTTTA	55	AF359979
v143	Lv10.201	GATCATCGCGGCAGTACG	59	AF359959
v145	Lv10.208	CCGTTTGGCGCTGCTTAG	55	AF359964
v150	Lv10.278	GCGTGTAATGCTTGCTGT	50	AF359980
v155	Lv10.343	TTTAGGACCTGCGGAGAA	52	AF359996

Abbreviations: T_a_: Annealing temperature ^a^ Most of the microsatellite are from Meehan et al. [18], however v110 is from Ball et al. [19].

Of the 600 polymorphic AFLP markers, 98 were segregated, of which 31 (32%) deviated from the expected Mendelian 3:1 ratios (P < 0.05). Of all the segregating markers (including SSR markers), 513 fitted in the expected segregation ratio and 47 markers showed a significant deviation from the 1:1 ratio (P < 0.05). Finally, 451 (87.91%) markers were grouped on the linkage map, leaving 63 (12.09%) markers unlinked.

### Linkage mapping

**Figure 1:**
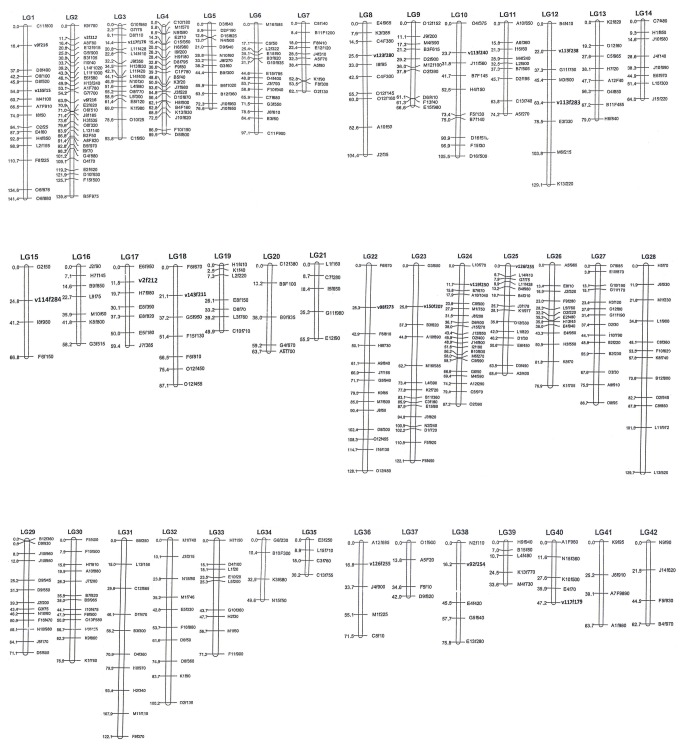
New linkage map in *L* *. vannamei* based on AFLP and SSR markers. Marker names are shown on the right and the adjacent marker spacing is displayed on the left in Kosambi centimorgans (cM). AFLP markers are in plain typeface and SSR markers are in bold.

A total of 600 AFLP markers and 25 SSR markers were employed to construct the linkage map. A total of 451 (34 distored markers) segregating markers were assigned to the growth linkage map (Figure 1). For linkage map, 451 (429 AFLPs, 22 SSRs) were assigned to 49 LGs (more than three markers), which covered 3313.9 cM (G_of_), in length with an average interval of 7.6 cM and a maximum interval of 32.8 cM. Length of LGs ranged from 13.1 cM (LG46) to 141.4 cM (LG1), and the number of markers per group varied from three to 28 with a mean of 8 (Table 3, Figure 1).

**Table 3:** Summary statistics of genetic linkage maps of *L* *. vannamei* .

	**Values (distored markers**)
Segregating markers	513 (47)
Number of markers in linkage analysis	505 (38)
Mapped markers	
AFLPs	429 (33)
SSRs	22 (1)
Unlinked doublets	6
Unlinked single markers	43 (4)
Linkage groups	49
Average number of markers per group	8
Minimum number of markers per group	3
Maximum number of markers per group	28
Average marker spacing (cM)	7.6
Maximum marker spacing(cM)	32.8
Minimum length of linkage group(cM)	13.1
Maximum length of linkage group(cM)	141.4
Observed genome length(cM)	
G_of_	3313.9
G_oa_	3627.6
Estimated genome length (cM)	
G_e1_	4543.0
G_e2_	4583.0
G_e_	4563.0
Genome coverage (%)	
C_of_	72.63
C_oa_	79.50

Abbreviations: G_of_: length of the framework map; G_oa_: total length considering all markers; G_e_: the estimated genome length that is calculated by the average of the two estimates(G_e1_ and G_e2_). C_of_ and C_oa_: observed genome coverages, determined by G_of_/G_e_ and G_oa_/G_e_.

### Genome estimation and map coverage

Assessed genome lengths through the two methods were 4543.0 cM and 4583.0 cM with an average of 4563.0 cM. Based on the observed length of the framework map (3313.9 cM) and the assessed genome length (4563.0 cM), the framework map had coverage of 72.63%. Map coverage increased to 79.50% if the doublets were included (Table 3).

### QTL analysis

**Figure 2:**
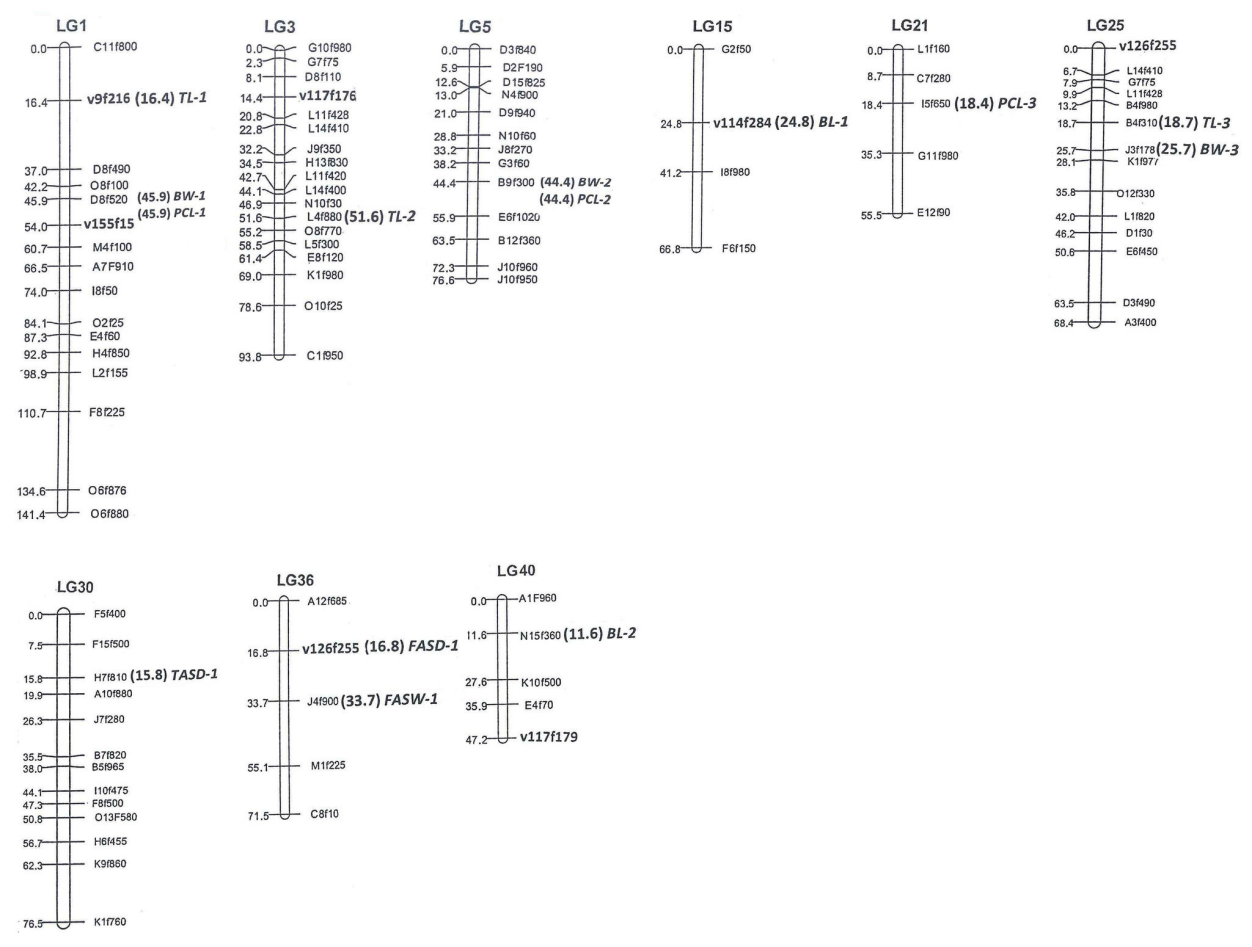
Putative QTLs for growth-related traits in *L* *. vannamei* . QTLs, Quantitative trait loci.

The profiles and characteristics of QTLs associated with seven traits are provided in Table 4, Figure 2. A total of 14 significant QTLs were detected on 9 LGs. Three QTLs (BW-1 to BW-3) were identified for body weight on three different LGs, each of these QTLs explained 2.62 to 18.34% of phenotypic variation individually. Three QTLs (TL-1 to TL-3) were identified for total length. Two QTLs (BL-1 and BL-2), accounting for 42.78-45.95% of phenotypic variation, were identified for body length. One QTL was identified for FASD, TASD and FASW, which respectively explained 61.42, 56.10 and 56.20% of phenotypic variation. Interestingly, three QTLs (PCL-1 to PCL-3) were detected for partial carapace length and accounted for 4.32 to 10.57% of phenotypic variation.

**Table 4:** Putative QTLs and their genetic effects for growth-related traits in *L* *. vannamei* .

**Traits**	**QTL**	**Linkage groups**	**Markers**	**Position**	***LOD* value**	**Additive effect**	**Variance (%**)
BW	BW-1	1	D8f520	45.90	3.79	71.12	15.86
	BW-2	5	B9f300	44.40	3.51	29.10	2.62
	BW-3	25	J3f178	25.70	4.44	73.21	18.34
TL	TL-1	1	v9f216	16.40	3.44	1.13	14.74
	TL-2	3	L4f880	51.60	4.16	2.12	10.96
	TL-3	25	B4f310	18.70	3.30	1.80	13.93
BL	BL-1	15	v114f284	24.80	3.99	2.80	45.95
	BL-2	40	N15f360	11.60	4.18	0.44	42.78
FASD	FASD-1	36	v126f255	16.80	4.69	0.50	61.42
TASD	TASD-1	30	H7f810	15.80	4.13	0.50	56.10
FASW	FASW-1	36	J4f900	33.70	4.23	0.50	56.20
PCL	PCL-1	1	D8f520	45.90	6.65	0.29	7.62
	PCL-2	5	B9f300	44.40	6.34	0.34	4.32
	PCL-3	21	I5f650	18.40	6.98	0.41	10.57

### Comparative mapping

The flanking sequences of AFLP loci placed on the map were used to compare with genomic DNA sequences of *P* *. japonicus* . Eight of which were significantly conserved between *L* *. vannamei* and *P* *. japonicus* (*E*-value < 1.0 × 10^-4^). The eight hits were distributed on eight of the 43 chromosomes in *P* *. japonicus* (Table 5). Unique correspondences were detected in four chromosome pairs (LG15- Chrom9, LG19- Chrom11, LG31- Chrom23, and LG35- Chrom19). Two AFLP loci (D8f520, M4f100) mapped on LG1 of *L* *. vannamei* at the locus of 45.9 cM and 60.7 cM were conserved in chromosome 1 and 17 of *P* *. japonicus* , respectively. Two AFLP loci (H13f830, J3f178) mapped on LG3 and LG25 in this research were both homologous with chromosome 25 of *P* *. japonicus* .

**Table 5:** Comparative chromosome of AFLP markers in linkage map of *L* *. vannamei* and *P* *. japonicus* .

**AFLP locus**	**Linkage group in *L* *. vannamei* **	**Chromosome in *P* *. japonicus* **
D8f520	LG1	Chrom1
M4f100	LG1	Chrom17
H13f380	LG3	Chrom25
J3f178	LG25	Chrom25
I8f980	LG15	Chrom9
E8f150	LG19	Chrom11
L13f150	LG31	Chrom23
C13f755	LG35	Chrom19

## Discussion

### Polymorphism and segregation of molecular markers

We constructed a genetic linkage map of *L*. *vannamei* with AFLP and SSR markers using a full-sib F_2_ intercross design. The relatively high yield of information achieved with AFLP markers makes it an efficient tool for mapping in shrimp. A total of 429 AFLP markers were identified in the mapping family using 100 *Eco*RI/*Mse*I primer combinations, with an average of 8 polymorphic bands per primer pairs. The average number of AFLP polymorphic markers produced from each primer combination was relatively lower than those in *P* *. monodon* [27], *P* *. japonicus* [8] and *L* *. vannamei* [9], but is almost similar to those generated 7.1 in *P* *. chinensis* [10], and 9.7 in *Portunustrituberculatus* [28]. This might be due to the differences among species or the possibility that the *L* *. vannamei* and *P* *. chinensis* used have low levels of diversity.

SSR markers have become markers of choice for construction of framework linkage maps due to their wide-ranging abundance, high polymorphism rate, Mendelian inheritance, and codominant expression [10]. Additionally, because microsatellites are sequence-tagged markers, integration with the linkage maps constructed by other laboratories or physical maps is feasible [29]. In this research, 22 of the 25 SSR markers (88%) were assigned to the framework map, which was higher than that in *P* *. chinensis* [10]. The transfer of SSRs will not only be useful for genetic mapping, but will also be critical for comparative mapping and evolution research within the genus *Penaeus* . With the progress of microsatellites for *L* *. vannamei* , they will increase the genome coverage, allowing construction of high-resolution linkage maps.

Segregation distortion among DNA markers has been observed to vary according to species and the nature of their mapping populations. Distorted segregation is a common observation in linkage analysis, and the rate of skewed loci in the *L* *. vannamei* linkage map (10%) is less than 12% in *P* *. chinensis* [8] and 16% of AFLPs in *Ictalurus* *punctatus* [11]. However, the percentage is higher than 8% of AFLPs and microsatellite markers in *Oreochromis* *niloticus* [30], and 8% of AFLPs in *C* *. virginica* [31]. The cause of segregation distortion in the linkage map of this research may be associated with the following two factors: (1) presence of genome structure differences between parents of mapping populations [32]; and (2) errors in marker genotyping. Besides these two factors, other research indicates that there is a biased selection of parental genotypes during F_2_ population development. Other reported explanations for the reason include loss of chromosomes [33], presence of gene conversion events [34], and homologous recombination that may cause segregation distortion [35].

### Linkage mapping

In the current map, newly developed SSR markers [10] were mapped in intervals of fragments with AFLPs to decrease the map distance between markers. Compared with the previous map [8], the genetic map presented in this research is improved, with an average genetic distance of 7.6 cM between adjacent markers and a maximum marker interval of 32.8 cM (LG45) (Figure 1). In contrast, Li et al. [8] reported that a total of 30 markers placed on maps with only two markers (m82.7 and m78.5) mapped into the targeted QTL LGs. Marker m82.7 was positioned in the QTL region of LG1 (103.8 cM), while marker m78.5 was in the middle of LG25 (outside of the suggestive QTL region of interest, 44.5 cM). Although we generally had very good coverage on map, one gap on LG45 was higher than 30 cM in terms of adjacent marker interval (Figure 1). The gap larger than 30 cM (32.8 cM on LG45) could correspond to the recombination of hot regions or marker scale regions. Compared to the average interval of 7.84 cM and 8.30 cM in the map reported by Li et al. [8], the map presented here is more saturated (7.6 cM). The map provides good coverage of the *L* *. vannamei* genome (79.50%).

LGs (49) obtained in the map developed in this research were higher than the chromosome number (n=44). This genome size is quite large, which may be a result of the many chromosomes of this species and chromosome interferences. The genetic map length should reflect the differences in recombination frequency; nevertheless, the total genetic map length (G_oa_) was 3627.6 cM and the estimated genome length (G_e_) was 4563.0 cM. This phenomenon could be attributed to several factors such as bias in collection of shrimp used for mapping population, number of markers used on map construction, and density and distribution of markers.

### QTL analysis

Genetic linkage maps allow a complete identification and the location of QTL for MAS and hence can be used in programs of genetic improvement in aquaculture. Physical maps enable the integration of linkage maps and karyotypes and are essential tools for comprehensive comparative genomic studies [36]. Moreover, the existence of a well-characterized physical map makes it more feasible to undertake a whole genome sequencing project [37]. In this work we reported the localization of growth-related genes, which showed statistically significant association with growth traits on *L* *. vannamei* chromosomes. A total of 14 QTLs were identified, including three QTLs for BW, three QTLs for TL, two QTLs for BL, three QTLs for PCL and one QTL for FASD, TASD and FASW.

For BW trait, the most dominant QTL (BW-3) was located on LG25, which explained 18.34% of phenotypic variation and indicated high additive effect (73.21), implying this locus should be a major genomic position controlling BW trait. For PCL trait, three major QTLs (PCL-1, PCL-2 and PCL-3) explained phenotypic variation ranging from 4.32 to 10.57% and showed all positive additive effect values, demonstrating that these three loci have great positive effect on PCL. Thus, it can be expected that high BW trait and short PCL trait could be well inherited in progeny if FG shrimp was used as female parent in breeding plans.

Numerous QTLs controlling different traits were clustered in very close interval of the same LG, for example, QTL TL-1 and BW-1 were identified in 16.4 and 45.9 cM interval on LG1; QTL BW-2 and PCL-2 were identified in the same chromosome region (44.4 cM) on LG5; QTL FASD-1 and FASW-1 were identified in 16.8 and 33.7 cM interval on LG36. The clustering of QTLs indicated the tight linkage of various genetic positions or the same chromosome region shared by many various QTLs. This tight linkage was confirmed by reported results from quantitative traits analysis [38]. Identification of QTLs influencing many traits could increase the efficiency of MAS and enhance genetic progress [39]. Meanwhile, the corresponding clustering of numerous QTLs affecting numerous traits provides an explanation for positive correlation among various traits.

### Comparative mapping

Comparative mapping between *L* *. vannamei* and *P* *. japonicus* can give us new insights into the evolution of shrimp species. In this paper, locus H13f380 was mapped on LG3 and locus J3f178 was mapped on LG25, however, they were both conserved with the same chromosome 25 of *P* *. japonicus* . It is speculated that LG3 and LG25 probably correspond to a single orthologous chromosome in *P* *. japonicus* .

Interestingly, locus D8f520 linked with a QTL affecting BW and PCL traits was conserved with a growth hormone gene of *P* *. monodon* (GenBank accession number: GO075401). This provides a new insight into the genetic base of QTL affecting BW and PCL.

In conclusion, the present map attains two goals: (1) the intermarker distance on this framework map and the good coverage provides enough marker density for mapping of quantitative traits; and (2) the map provides an effective tool for genetic analysis and manipulations. However, this map needs further improvement and QTLs also needs further precise location, but it will still be a very useful tool for shrimp genetic research and selective breeding schemes in the future. Combining next generation sequencing and genotyping technological advances, more markers could be added to genetic map of shrimp to improve the genetic map quality. Further research of growth-related traits will help us expand our knowledge of crustacean growth and to produce high-quality shrimp products.
