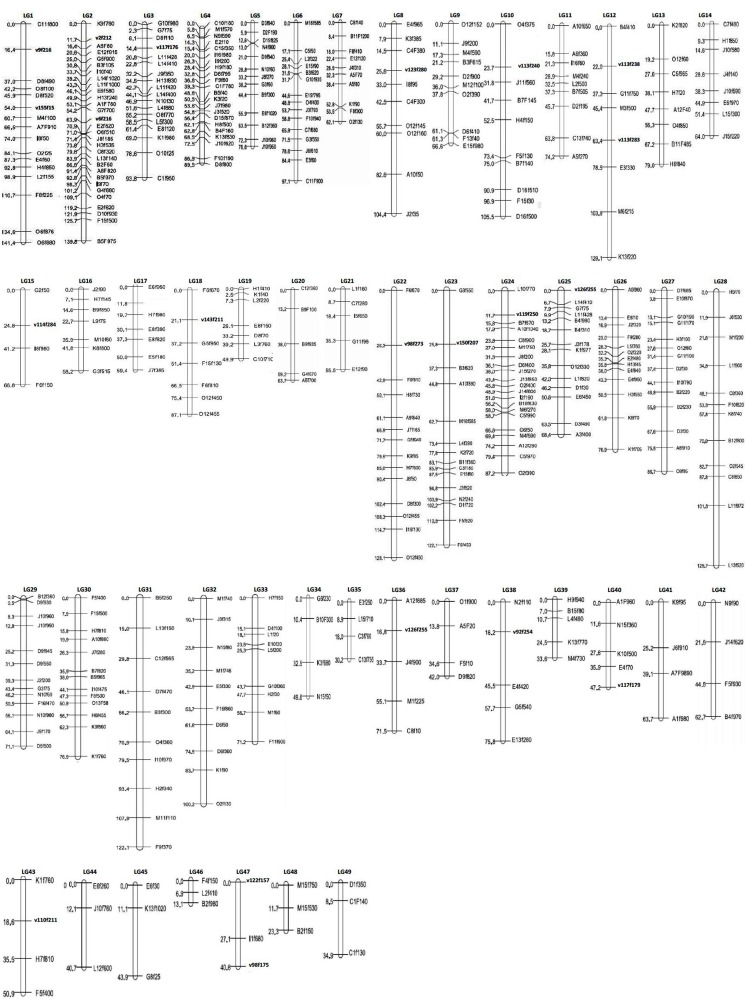# Correction: Genetic Map Construction and Quantitative Trait Locus (QTL) Detection of Growth-Related Traits in *Litopenaeus vannamei* for Selective Breeding Applications

**DOI:** 10.1371/annotation/80183e4b-b1b4-48cc-b307-865af97df0ce

**Published:** 2013-10-10

**Authors:** Farafidy Andriantahina, Xiaolin Liu, Hao Huang

There was information missing from Figure 1. A correct, complete version of the figure is available here: 

**Figure:**